# Silibinin Restores NAD^+^ Levels and Induces the SIRT1/AMPK Pathway in Non-Alcoholic Fatty Liver

**DOI:** 10.3390/nu9101086

**Published:** 2017-09-30

**Authors:** Federico Salomone, Ignazio Barbagallo, Justyna Godos, Vincenzo Lembo, Walter Currenti, Diana Cinà, Roberto Avola, Nicolantonio D’Orazio, Filomena Morisco, Fabio Galvano, Giovanni Li Volti

**Affiliations:** 1 Division of Gastroenterology, Acireale Hospital, Azienda Sanitaria Provinciale di Catania, 95124 Catania, Italy; 2 Department of Drug Sciences, University of Catania, 95125 Catania, Italy; ignazio.barbagallo@unict.it; 3 Department of Biomedical and Biotechnological Sciences, University of Catania, 95123 Catania, Italy; justynagodos@gmail.com (J.G.); currentiw@gmail.com (W.C.); ravola@unict.it (R.A.); fgalvano@unict.it (F.G.); livolti@unict.it (G.L.V.); 4 Department of Clinical Medicine and Surgery, University of Naples “Federico II”, 80131 Naples, Italy; v.lembo@hotmail.it (V.L.); filomena.morisco@unina.it (F.M.); 5 Division of Laboratory Medicine, Hospital “Garibaldi”, 95124 Catania, Italy; dianacinact@gmail.com; 6 Department of Medical, Oral and Biotechnological Sciences, University of Chieti, 66013 Chieti, Italy; nicolantonio.dorazio@unich.it

**Keywords:** silibinin, NAD^+^, SIRT1, AMPK, lipogenesis

## Abstract

Nicotinamide adenine dinucleotide (NAD^+^) homeostasis is emerging as a key player in the pathogenesis of non-alcoholic fatty liver disease (NAFLD) and is tightly linked to the SIRT1/5’-AMP-activated protein kinase (AMPK) pathway. Silibinin, the main component of silymarin, has been proposed as a nutraceutical for the treatment of NAFLD. In this study, we aimed to identify whether silibinin may influence the NAD^+^/SIRT1 axis. To this end, C57BL/6 mice were fed a high fat diet (HFD) for 16 weeks, and were treated with silibinin or vehicle during the last 8 weeks. HepG2 cells were treated with 0.25 mM palmitate for 24 h with silibinin 25 µM or vehicle. HFD and palmitate administration led to oxidative stress, poly-(ADP-ribose)-polymerase (PARP) activation, NAD^+^ consumption, and lower SIRT1 activity. In mice fed the HFD, and in HepG2 treated with palmitate, we consistently observed lower levels of phospho-AMPK^Thr172^ and phospho-acetyl-CoA carboxylase^Ser79^ and higher levels of nuclear sterol regulatory element-binding protein 1 activity, indicating de novo lipogenesis. Treatment of mice and HepG2 with silibinin abolished oxidative stress, and inhibited PARP activation thus restoring the NAD^+^ pool. In agreement with preserved NAD^+^ levels, SIRT1 activity and AMPK phosphorylation returned to control levels in mice and HepG2. Our results further indicate silibinin as a promising molecule for the treatment of NAFLD.

## 1. Introduction

Non-alcoholic fatty liver disease (NAFLD) represents the most common liver disease in industrialized countries [1]. Epidemiological studies including prospective cohorts have shown that liver fat accumulation per se precedes the onset of insulin resistance and metabolic syndrome [2]. Beyond metabolic complications, a subset of patients with NAFLD develop non-alcoholic steatohepatitis (NASH), which is emerging as a leading cause of liver transplantation due to progression to cirrhosis and cancer [3]. The most effective therapeutic option to prevent onset and progression of NAFLD is a healthy lifestyle [4], although only few patients are compliant with lifestyle changes. For this reason, several drugs have been proposed so far for counteracting progression of fatty liver to inflammation and fibrosis, but with unsatisfactory results [5]. Recent translational studies have indicated that natural phenolic compounds may display beneficial effects in NAFLD by modulating hepatic lipid homeostasis [6].

Nicotinamide adenine dinucleotide (NAD^+^) is a co-enzyme involved in redox reactions that plays a protective role against metabolic diseases and aging [7,8]. The beneficial effects of NAD^+^ can be in part attributed to the activity of the NAD-dependent protein deacetylase sirtuin-1 (SIRT1), an enzyme that regulates metabolic homeostasis by deacetylating lysine residues on histones and transcriptional regulators [9]. SIRT1 activates 5’-AMP-activated protein kinase (AMPK) [10] which controls energy expenditure in health and disease [11]. NAD^+^ levels depend on the balance between its biosynthesis and its consumption by several NAD-dependent enzymes, especially poly-(ADP-ribose)-polymerases (PARPs), a family of enzymes that protect cells against oxidative DNA damage [12]. However, although PARPs can be considered as genome guardians, hyperactivation of PARPs leads to NAD^+^ depletion and thus cell injury [13]. Recent evidence indicates that NAD^+^ repletion reverses fatty liver in mice by restoring SIRT1 activity and modulating the mitochondrial efficiency [14,15].

Silibinin is a flavonolignan that constitutes the main component of silymarin, a lipophilic milk thistle extract, which is consumed worldwide as a nutraceutical for liver diseases [6]. Interest in milk thistle derivatives for the treatment of NAFLD is mirrored by randomized controlled trials on efficacy and safety (www.clinicaltrials.gov). Traditionally, silymarin has been characterized by the potent anti-oxidant activity of its main component silibinin [16]. Recently, beyond antioxidant effects, it was showed that silibinin favourably modulates lipid homeostasis and insulin sensitivity in several models of NAFLD [6]. In this study, we aimed to establish whether silibinin may influence NAD^+^ homeostasis and the SIRT1/AMPK pathway in a mouse model of fatty liver and in an in vitro model of hepatocyte lipotoxicity.

## 2. Materials and Methods

### 2.1. Cell Culture

The HepG2 cells (American Type Culture Collection, Manassas, VA, USA) were a kind gift from Prof. Maurizio Parola of the University of Torino. Cell viability measurement was performed using a Muse™ Cell Analyzer (Merck Millipore, Milan, Italy) according to the manufacturer’s instructions. Treatment of HepG2 with the fatty acid (FA) palmitate was performed as previously described [17]. Briefly, cells at low passages were cultured in DMEM (Sigma-Aldrich, Milan, Italy) supplemented with 10% FBS (EuroClone, Milan, Italy), 100 U/mL penicillin (Life Technologies, Milan, Italy) and 100 µg/mL streptomycin (Life Technologies) at 37 °C in a humidified incubator in a 95% air and 5% CO_2_ atmosphere [18]. Upon reaching 80–90% confluence, the HepG2 cells were incubated for 24 h as follows: HepG2 + vehicle (Bovine Serum Albumin (BSA) 5% + DMSO); HepG2 + FA (BSA 5% + DMSO + palmitate 0.25 mM); HepG2 + FA + silibinin (BSA 5% + DMSO + palmitate 0.25 mM + silibinin 25 µmol/L). Silibinin (Indena, Milan, Italy) was dissolved in DMSO at room temperature and administered at a 25 µmol/L concentration. This dose of silibinin was chosen on the basis of previous studies [18,19].

### 2.2. Animals

All procedures fulfilled the Italian Guidelines for the Use and Care of Laboratory Animals. Eight- to ten-week-old C57BL/6 mice (Charles River Labs, Lecco, Italy) were maintained in a temperature- and light-controlled facility. Animals were fed either a standard diet (SD) or a high fat diet (HFD) for 16 weeks. During the last 8 weeks of the HFD, mice were randomly selected to be daily administered silibinin (Indena, Milan, Italy) by gavage at a dose of 5 mg/kg of body weight or vehicle. Silibinin, as powder, was dissolved in DMSO. Diets were obtained from Harlan Teklad (Madison, WI, USA). The SD provided 3.3 kcal/g with 60% carbohydrates, 23% proteins, and 17% fat. The HFD provided 5.2 kcal/g with 60% fat, 20% proteins, and 20% carbohydrates. Mice were distributed in three groups: group I included six mice fed the SD (SD); group II comprised eight mice fed the HFD (HFD); group III comprised eight mice fed the HFD and treated with silibinin (HFD + SIL. During the study period, food and beverage consumption was recorded twice weekly; body weight was recorded weekly. After sacrifice by CO_2_ asphyxiation, blood and liver samples were obtained, processed, and stored at −80 °C for successive molecular determinations. Ethics approval was obtained from the University of Catania.

### 2.3. Oil Red O and Hematoxylin/Eosin Staining

Lipid accumulation in HepG2 cells was assessed by Oil Red O staining. In brief, cells were fixed in 4% paraformaldehyde for 30 min, followed by staining with Oil Red O for 30 min. Next, fixed cells were gently washed with isopropanol, and then three times washed with distilled water. Results were determined by fluorescence microscopy imagining and by measurement of absorbance at 500 nm using Synergy HT (BioTek, Milan, Italy). The cells were cultured in 24-well flasks. Six wells were used for each treatment and the experiment was repeated three times.

Liver histopathology was performed on 5 µm liver slices stained with hematoxylin and eosin by standard procedures. Liver slices were examined without knowledge of the type of treatment undergone.

### 2.4. Biochemical Analyses

Sandwich ELISA for threonine 172-phosphorylated AMPKα (#7959), total AMPKα (#7961), serine 79-phosphorylated acetyl-CoA carboxylase (ACC) (#7986), and total ACC (#7996) were purchased from Cell Signalling Technology (Beverly, MA, USA). Absorbance was read at 450 nm.

PARP activity was assayed using the universal colorimetric PARP assay kit (Trevigen Inc., Gaithersburg, MD, USA) according to the manufacturer’s instructions. SIRT1 activity was measured using the SIRT1 Fluorometric Drug Discovery Kit (ENZO Life Sciences Inc., Farmingdale, NY, USA) according to the manufacturer’s instructions. Changes in PARP1 and SIRT1 activities were calculated against the mean value of PARP1 and SIRT1 activities in control liver and cells, and expressed as percent of control. NAD^+^ levels were measured using EnzyChrom™ NAD^+^/NADH Assay kit (BioAssay Systems, Hayward, CA, USA) according to the manufacturer’s instructions and as previously described [20]. Sterol regulatory element-binding protein 1 (SREBP-1) activity was measured using a SREBP-1 transcription factor assay kit (Abcam, Cambridge, UK), according to manufacturer’s instruction, on nuclear extracts of HepG2 that were obtained by a nuclear extraction kit (Abcam, Cambridge, UK).

Triglyceride content in HepG2 and mouse liver samples was measured using a colorimetric assay according to the manufacturer’s instructions (Biovision, Mountain View, CA, USA).

### 2.5. Real-Time PCR

Gene expression was assessed by quantitative real-time polymerase chain reaction (qRT-PCR). Reverse transcription reaction was carried out on 1 µg of total RNA using oligo (dT) primers (Table S1) and MultiScribe™ Reverse Transcriptase (Applied Biosystems, Milan, Italy), according to the vendors’ instructions. Quantitative RT-PCR was performed in a 7900 HT Fast Start real-time PCR system (Applied Biosystems) in a mixture containing SYBR^®^ Green PCR Master Mix (Life Technologies), specific primers, and 50 ng of cDNA in a total volume of 20 µL. The GAPDH housekeeping gene was used as a reference. The ΔCt protocol was used to determine the absolute values of gene expression.

### 2.6. ROS (Reactive Oxygen Species) Measurement

Reactive Oxygen Species (ROS) production in HepG2 was measured using a Muse™ Cell Analyzer (Merck Millipore) according to the manufacturer’s instructions. The Muse^®^ Oxidative Stress Kit (Merck Millipore) allows for the quantitative measurement of Reactive Oxygen Species, namely superoxide radicals in cells undergoing oxidative stress. ROS generation in liver homogenates was determined by using 2′,7′-dichlorodihydrofluoroscein diacetate as a probe. Dichlorodihydrofluorescein diacetate formation was determined fluorometrically with a Hitachi F-2000 (Hitachi Ltd., Tokyo, Japan) fluorescence spectrophotometer at excitation wavelength of 488 nm and emission wavelength of 525 nm at 37 °C.

### 2.7. Statistical Analysis

Data are presented as mean ± standard error of mean (SEM). Each determination was performed at least in triplicate. Significance was assessed by ANOVA followed by Bonferroni test; *p* < 0.05 was considered to be statistically significant. Statistical analysis was performed using GraphPad Prism (San Diego, CA, USA).

## 3. Results

### 3.1. Silibinin Reduces Lipid Accumulation in HepG2 and Mice Liver

**Figure 1:**
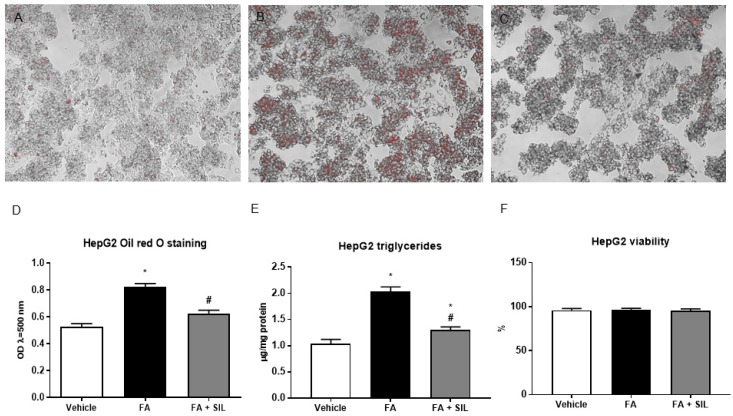
Effects of silibinin on steatosis in HepG2. (**A**–**D**), Following a 24-h culture with 0.25 mM palmitate, HepG2 + vehicle displayed lipid droplet accumulation, as shown by Oil Red O staining. In cells treated with 25 µM silibinin, a reduction of lipid accumulation was observed. The cells were cultured in 24-well flasks. Six wells were used for each treatment and the experiment was repeated three times; (**E**) Consistently with Oil Red O staining, triglyceride content was reduced in HepG2; (**F**) Palmitate or silibinin didn’t affect cell viability, assessed using a Muse™ Cell Analyzer. All values are expressed as mean ± SEM of three experiments (*n* = 3) in duplicate. * *p* < 0.05 vs. vehicle; ^#^ *p* < 0.05 vs. FA. FA: Fatty acid; OP: Optical density; SIL: Silibinin.

**Figure 2:**
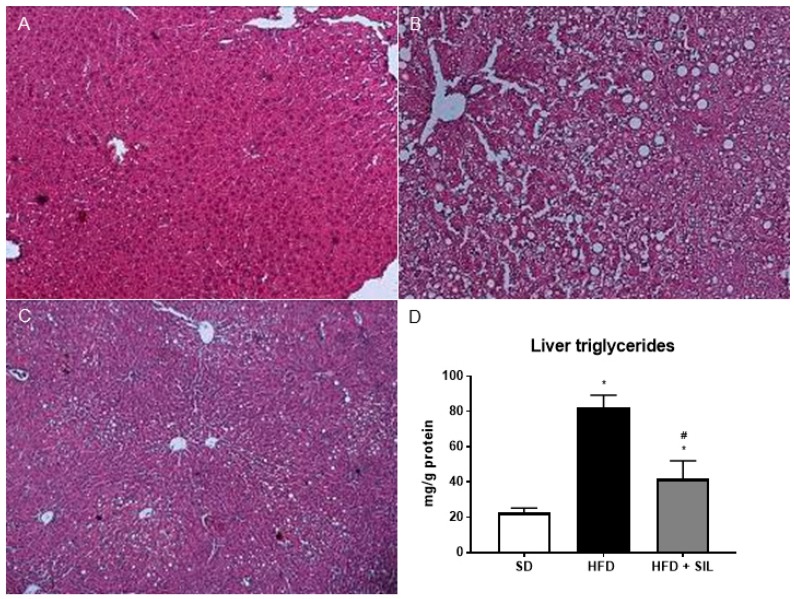
Effects of silibinin on liver steatosis in mice. Liver sections were stained with hematoxylin and eosin. (**A**) Liver sections of mice fed the SD; (**B**) Following a 16-week HFD, the mouse livers displayed microvescicular steatosis with a panacinar pattern; (**C**) An 8-week treatment with silibinin attenuated steatosis; (**D**) Consistently with in vitro results, liver triglyceride content in mice treated with silibinin, as assessed by a colorimetric assay, was markedly reduced. * *p* < 0.05 vs. SD; ^#^ *p* < 0.05 vs. HFD. HFD: High fat diet; SD: Standard diet; SIL: Silibinin.

A 24-h culture with 0.25 mM palmitate led to lipid droplet accumulation in HepG2, without affecting cell viability (Figure 1). In cells treated with 25 µM silibinin, lipid accumulation was markedly reduced (Figure 1). Liver sections from mice fed the HFD for 16 weeks showed microvescicular steatosis with a panacinar pattern (Figure 2). By contrast, silibinin treatment during the last 8 weeks of the HFD attenuated steatosis (Figure 2). Consistently with in vitro results, liver triglyceride content was markedly decreased in mice treated with silibinin (Figure 2). Interestingly, these effects on liver steatosis occurred despite no significant effects on body weight (Figure S1).

### 3.2. Silibinin Restores NAD^+^ Levels and Induces SIRT1/AMPK Signaling

**Figure 3:**
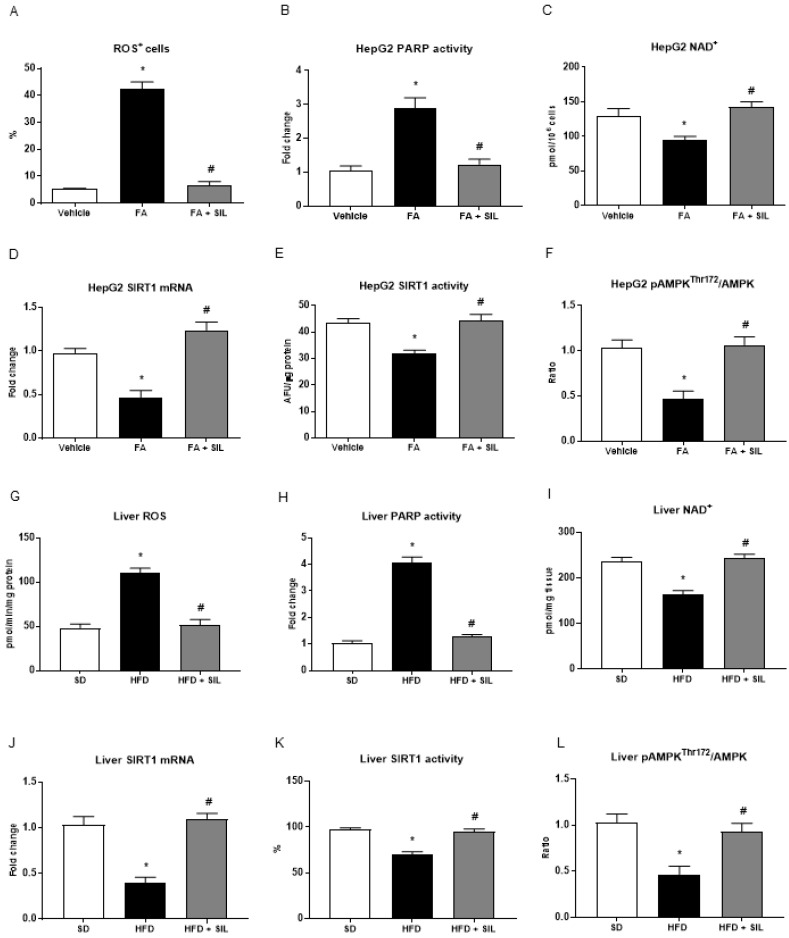
Effects of silibinin on ROS, PARP, NAD^+^, SIRT1 and AMPK levels in HepG2 and mice liver (**A**–**C**) Palmitate administration led to higher levels of ROS and PARP activity and lower levels of NAD^+^. Treatment of HepG2 with 25 Mm silibinin abolished oxidative stress and inhibited PARP activation, thus restoring NAD^+^ levels; (**D**–**E**) SIRT1 activity and mRNA expression were lower in HepG2 treated with 0.25 mM palmitate compared with control cells; (**F**) Levels of phosphorylated and total AMPKα were measured by sandwich ELISA and the ratio is shown; treatment with silibinin restored SIRT1 activity and expression and induced AMPK phosphorylation at Thr172; (**G**–**L**) In vitro results were confirmed in mice fed a 16-week HFD and treated with silibinin during the last 8 weeks. All values are expressed as mean ± SEM of three experiments (*n* = 3) in duplicate. * *p* < 0.05 vs. vehicle; ^#^ *p* < 0.05 vs. FA. AFU: Arbitrary fluorescence units; AMPK: AMP-kinase; FA: Fatty acid; HFD: High fat diet; NAD^+^: Nicotinamide adenine dinucleotide; PARP: poly-(ADP-ribose)-polymerase; ROS: Reactive oxygen species; SD: Standard diet; SIL: Silibinin.

Based on steatosis results, we aimed to verify the hypothesis that silibinin may influence NAD^+^ levels and the SIRT1/AMPK pathway. As expected, palmitate administration increased the mean number of ROS^+^ cells and enhanced PARP activity leading to lower NAD^+^ levels in HepG2 cells (Figure 3). Similarly, the HFD induced oxidative stress and higher PARP activity while lowering the NAD^+^ pool in mouse livers (Figure 3). As a consequence of PARPs activation and NAD^+^ consumption, palmitate administration reduced the expression and activity of SIRT1 (Figure 3). The ratio between the levels of AMPKα phosphorylated at the threonine 172 residue and total AMPKα was consistently lower in HepG2 treated with the FA + vehicle compared with the control cells (Figure 3).

Treatment of HepG2 and mice with silibinin abolished oxidative stress and inhibited PARP activation, thus restoring NAD^+^ levels (Figure 3). In agreement with preserved NAD^+^ levels, SIRT1 activity and AMPK phosphorylation returned to control levels in HepG2 and in mice (Figure 3).

### 3.3. Silibinin Inhibits De Novo Lipogenesis and Promotes Mitochondrial β-Oxidation

**Figure 4:**
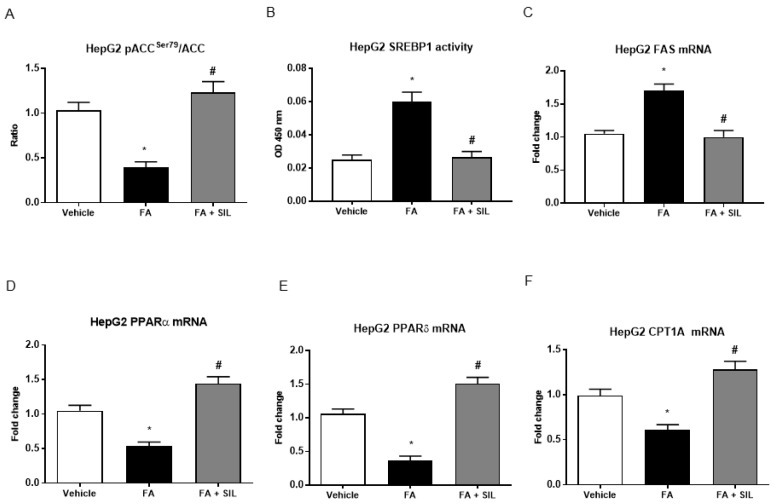
Effects of silibinin on de novo lipogenesis and lipolysis in HepG2. (**A**–**F**) HepG2 exposed to palmitate displayed lower levels of phosphorylation at the serine 79 residue of ACC, the rate-limiting enzyme of de novo lipogenesis. A 24 h culture with palmitate was also consistently associated with higher transcriptional activity of SREBP-1, the main transcription factor promoting lipogenesis, and higher levels of FAS, a downstream target of SREPB-1. Treatment with silibinin stimulated the phosphorylation of ACC, leading to its inactivation and thus to decreased conversion of acetyl-CoA to malonyl-CoA. It also reduced the transcriptional activity of SREBP-1, assessed on nuclear extracts, thus down-regulating the expression of FAS. Concomitantly with the effects on lipogenesis, we observed that treatment with silibinin up-regulated the expression of CPT1A, the rate limiting enzyme of mitochondrial β-oxidation. All values are expressed as mean ± SEM of three experiments (*n* = 3) in duplicate. * *p* < 0.05 vs. vehicle; ^#^ *p* < 0.05 vs. FA. ACC: acetyl Co-A carboxylase; CPT1A: carnitine palmitoyltransferase 1A; FA: Fatty acid; FAS: Fatty acid synthase; PPARα: Peroxisome proliferator-activated receptor alpha; PPARδ: Peroxisome proliferator-activated receptor delta; SREBP-1: Sterol regulatory element-binding protein 1; SIL: Silibinin.

A 24-h exposure of HepG2 to palmitate led to lower levels of phosphorylation at the serine 79 residue of ACC (Figure 4), the rate-limiting enzyme of de novo lipogenesis [21], which is a direct downstream target of AMPK [22]. Because of AMPKα phosphorylation and activation, cells treated with silibinin displayed an increase in levels of phospho-ACC, indicating lower ACC activity (Figure 4). As expected, a 24 h culture with palmitate was also associated with a higher transcriptional activity of SREBP-1, the main transcription factor promoting lipogenesis [23]. Concomitantly with the phosphorylation of ACC, we observed that silibinin administration reduced the activity of SREBP-1 in nuclear extracts of HepG2 (Figure 4). The expression of fatty acid synthase (FAS), a downstream target of SREBP-1 [23], was similarly down-regulated by silibinin (Figure 4).

**Figure 5:**
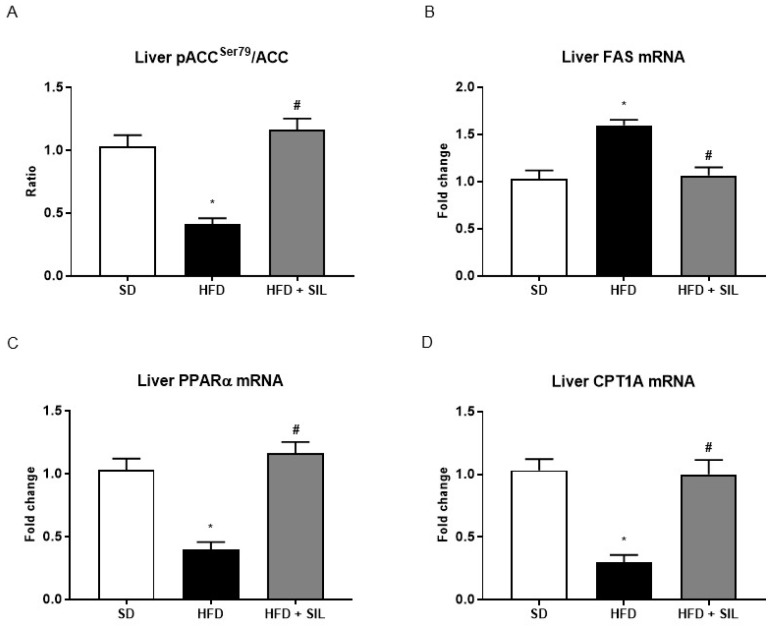
Effects of silibinin on hepatic de novo lipogenesis and lipolysis in mice. (**A**–**D**) Levels of phosphorylated and total ACC, the rate-limiting enzyme of de novo lipogenesis, were measured by sandwich ELISA and the ratio is showed. Phosphorylation at the serine 79 residue inhibits ACC activity, leading to decreased conversion of acetyl-CoA to malonyl-CoA. Following a 16-week HFD, mice displayed lower levels of hepatic phospho-ACC and higher levels of FAS, a main lipogenic enzyme. Treatment with silibinin induced ACC phosphorylation at Ser79 and down-regulated FAS expression. Consistently with these results, gene expression of CPT1A, the rate limiting enzyme of mitochondrial β-oxidation, and gene expression of PPARα, its upstream transcription factor, were downregulated in the livers of obese mice. Concomitantly with the effects on lipid accumulation, we observed that treatment with silibinin up-regulated the expression of PPARα and of CPT1A. All values are expressed as mean ± SEM of three experiments (*n* = 3) in duplicate. * *p* < 0.05 vs. SD; ^#^ *p* < 0.05 vs. HFD. ACC: Acetyl-coA carboxylase; CPT1A: Carnitine O-palmitoyltransferase 1A; FAS: Fatty acid synthase; HFD: High fat diet; PPARα: Peroxisome proliferator-activated receptor alpha; SD: Standard diet; SIL: Silibinin.

Phosphorylation and inactivation of ACC leads to decreased conversion of acetyl-CoA to malonyl-CoA [23], which is an allosteric inhibitor of mitochondrial carnitine palmitoyltransferase 1A (CPT1A), the enzyme responsible for transport of acyl-CoAs into mitochondria for oxidation [24]. In accordance with the inhibition of ACC activity by AMPK and thus with the presumably reduced levels of malonyl-CoA, we found that silibinin administration increased gene expression of CPT1A (Figure 4). Consistently with lower levels of CPT1A, the gene expression of PPAR-α, the nuclear transcription factor of CPT1A [25], was reduced in HepG2 treated with FA alone and was augmented in cells treated with silibinin (Figure 4). Furthermore, culture of HepG2 with palmitate also led to reduced mRNA levels of PPAR-δ, a main transcription factor promoting FA oxidation in the liver [24], whereas treatment with silibinin also up-regulated PPAR-δ expression (Figure 4). Similarly to what was observed in vitro, we found that, in mice fed the HFD, treatment with silibinin led to the inhibition of lipogenesis and the induction of β-oxidation in the liver (Figure 5).

## 4. Discussion

In this study, we aimed to investigate whether silibinin, a flavonolignan contained in milk thistle, may exert hepatoprotective effects by modulating NAD^+^ homeostasis and the SIRT1/AMPK pathway. In addition to its established protective roles in the context of diabetes and obesity [7,8], NAD^+^ homeostasis has been recently shown to play an important role in the onset of fatty liver by modulating mitochondrial efficiency [14]; consistently, it has been showed that AMPK regulation of mitochondrial function may be crucial in the progression of human steatohepatitis [25]. AMPK activation requires the activity of SIRT1 [26], which controls hepatic lipid metabolism through modulation of PPARs activity [27,28]. In this study, we showed for the first time that silibinin, a potent natural antioxidant, restores NAD^+^ levels and induces the SIRT1/AMPK pathway in vitro and in vivo. It is plausible that these effects may be in part dependent on silibinin antioxidant capacity. Overall, we may speculate that lower oxidative stress leads to lower PARPs activation, thus leading to lower NAD^+^ consumption. In line with the co-regulation of NAD^+^ and SIRT1, a sufficient NAD^+^ pool in turn maintains metabolic health by restoring SIRT1 activity and signaling. This may be a common mechanism that explains why several flavonoids are able to inhibit PARPs, elevate intracellular NAD^+^ levels, and activate NAD^+^-dependent sirtuin-mediated signaling pathways in metabolic and age-related chronic diseases [29]. Nonetheless, since it has been demonstrated that SIRT1 activity is also directly inhibited by oxidative stress [30], we may also speculate that the well-established antioxidant activity of silibinin may also directly influence SIRT1 activity by modulating the redox *status*. Interestingly, we also found that under lipotoxic conditions silibinin induces the expression of PPAR-α and PPAR-δ in HepG2. These last results appear relevant because Elafibranor, a synthetic PPAR-α/δ agonist, was recently shown to be effective in experimental and human NAFLD [31,32]. However, functional studies are needed to establish if silibinin acts as a direct PPAR-α/δ agonist.

It should be underlined that our novel results add to a growing body of experimental evidence showing the beneficial metabolic effects of silibinin in different models of NAFLD [33,34,35,36,37,38]. Interestingly, some studies have showed that silibinin may influence not only steatosis mechanisms but also mechanisms related to liver inflammation and fibrosis. Our group showed that silibinin inhibits the activity of NF-κB and down-regulates the expression of its pro-inflammatory targets in mice with genetic and diet-induced NAFLD [37,38]. Moreover, silibinin suppresses in vitro activation of rat Kupffer cells [39] and human hepatic stellate cells [19,40]. In this respect, although several SIRT1/AMPK activators have been proposed for NAFLD treatment [41,42], we think that silibinin can be more effective than the others because it can beneficially modulate almost all the molecular mechanisms associated with onset and progression of NAFLD [43].

Besides the experimental data obtained so far, pilot clinical trials have assessed the efficacy of specific nutraceuticals containing silibinin. In a randomized controlled trial of biopsy-proven NAFLD patients, Loguercio et al. showed that a 200 mg daily dose of silibinin not only decreases serum enzymes and insulin resistance, but also led to improvement of histological scores of steatosis, inflammation, and fibrosis [44]. Recently, a small randomized trial showed that supplementation of a 540 mg daily dose of silymarin for three months has better effects on liver steatosis, assessed by non-invasive score, than lifestyle changes [45]. However, translational approaches are hampered by the issue of bioavailability in regards to the plethora of oral nutraceuticals containing silibinin [46]. Thus, the “pharmaceutical” design of a silibinin-based nutraceutical in order to allow adequate bioavailability remains a priority.

## 5. Conclusions

In conclusion, in this study we provided the first evidence that silibinin restores NAD^+^ levels and induces the SIRT1/AMPK pathway in vitro and in vivo. Our results further suggest that silibinin may be a promising molecule for the treatment of NAFLD.

## Supplementary Materials

The following are available online at www.mdpi.com/2072-6643/9/10/1086/s1, Figure S1: Final body weight, Table S1: Primers sequences used for real time PCR analysis.
