# Supplementary material for: Silibinin Restores NAD+ Levels and Induces the SIRT1/AMPK Pathway in Non-Alcoholic Fatty Liver
Source: Nutrients. 2017 Sep 30;9(10):1086. doi: 10.3390/nu9101086 (PMC5691703; doi:10.3390/nu9101086)
Supplement: Supplementary file 1 [file nutrients-09-01086-s001.zip › nutrients-215033-supplementary.pdf]

Supplementary Table S1. Primers sequences used for real time PCR analysis.

Gene name	Forward	Reverse
hGAPDH	5′-AGACACCATGGGGAAGGTGA-3′	5′-TGGAATTTGCCATGGGTGGA-3′
hCPT1A	5′-TCCAGTTGGCTTATCGTGGTG-3′	5′-TCCAGAGTCCGATTGATTTTTGC-3′
hFAS	5′-CGGAGGCATCAACCCAGATT-3′	5′-GATGGTGGTGTAGACCTTCCG-3′
hPPARα	5′-AAGAGCTTGGAGCTCGGC-3′	5′-TGAAAGCGTGTCCGTGATGA-3′
hPPARδ	5′-AGAGTACGTGGGAGAAATGAC-3′	5′-GATGGCCACCTCTTTGCTCT-3′
hSIRT1	5′-TGATTGGCACAGATCCTCGAA-3′	5′-AAGTCTACAGCAAGGCGAGC-3′
mβ-actin	5′-TGTCCACCTTCCAGCAGATGT-3′	5′-AGCTCAGTAACAGTCCGCCTAGA-3′
mCPT1A	5′-TGAGCAACAAGCTGAAGTCC-3′	5′-AGGCACCTCCAGTCACACAA-3′
mFAS	5′-TTCAGCACAGTGGATCATCA-3′	5′-CAAGTAATAAGCACTCCTGA-3′
mPPARα	5′-GCGCAAGTGTTACGAAGTGG-3′	5′-GGTTGGCAGCTCTCATGTCT-3′
mSIRT1	5′-AGAACCACCAAAGCGGAAA-3′	5′-TCCCACAGGAGACAGAAACC-3′

Supplementary Figure S1.

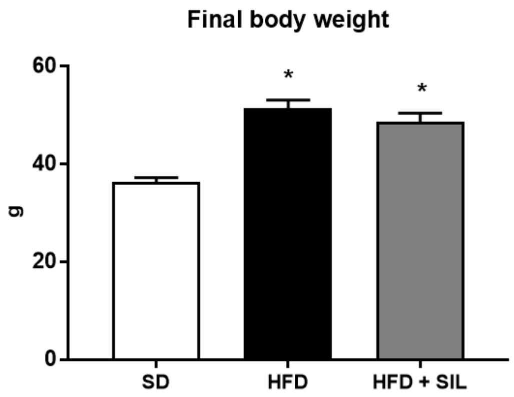

* $p < 0.05$ vs. SD
